# Analysis of the Role of CX3CL1 (Fractalkine) and Its Receptor CX3CR1 in Traumatic Brain and Spinal Cord Injury: Insight into Recent Advances in Actions of Neurochemokine Agents

**DOI:** 10.1007/s12035-016-9787-4

**Published:** 2016-03-01

**Authors:** Łukasz A. Poniatowski, Piotr Wojdasiewicz, Maciej Krawczyk, Dariusz Szukiewicz, Robert Gasik, Łukasz Kubaszewski, Iwona Kurkowska-Jastrzębska

**Affiliations:** 1 Department of General and Experimental Pathology, 2nd Faculty of Medicine, Medical University of Warsaw, Pawińskiego 3C, 02-106 Warsaw, Poland; 2 Department of Rheumaorthopaedics, Eleonora Reicher National Institute of Geriatrics, Rheumatology and Rehabilitation, Spartańska 1, 02-637 Warsaw, Poland; 3 Department of Neuroorthopaedics and Neurology, Eleonora Reicher National Institute of Geriatrics, Rheumatology and Rehabilitation, Spartańska 1, 02-637 Warsaw, Poland; 4 2nd Department of Neurology, Institute of Psychiatry and Neurology, Sobieskiego 9, 02-957 Warsaw, Poland; 5 Department of Pediatric and Neurological Rehabilitation, Faculty of Rehabilitation, Józef Piłsudski University of Physical Education, Marymoncka 34, 00-968 Warsaw, Poland; 6 Department of Orthopaedics and Traumatology, Wiktor Dega Orthopaedic and Rehabilitation Clinical Hospital, Poznań University of Medical Sciences, 28 Czerwca 1956 135/147, 61-545 Poznań, Poland

**Keywords:** CX3CL1, Fractalkine, CX3CR1, Chemokines, Traumatic brain injury, Spinal cord injury, Neuroinflammation

## Abstract

CX3CL1 (fractalkine) is the only member of the CX3C (delta) subfamily of chemokines which is unique and combines the properties of both chemoattractant and adhesion molecules. The two-form ligand can exist either in a soluble form, like all other chemokines, and as a membrane-anchored molecule. CX3CL1 discloses its biological properties through interaction with one dedicated CX3CR1 receptor which belongs to a family of G protein-coupled receptors (GPCR). The CX3CL1/CX3CR1 axis acts in many physiological phenomena including those occurring in the central nervous system (CNS), by regulating the interactions between neurons, microglia, and immune cells. Apart from the role under physiological conditions, the CX3CL1/CX3CR1 axis was implied to have a role in different neuropathologies such as traumatic brain injury (TBI) and spinal cord injury (SCI). CNS injuries represent a serious public health problem, despite improvements in therapeutic management. To date, no effective treatment has been determined, so they constitute a leading cause of death and severe disability. The course of TBI and SCI has two consecutive poorly demarcated phases: the initial, primary injury and secondary injury. Recent evidence has implicated the role of the CX3CL1/CX3CR1 axis in neuroinflammatory processes occurring after CNS injuries. The importance of the CX3CL1/CX3CR1 axis in the pathophysiology of TBI and SCI in the context of systemic and direct local immune response is still under investigation. This paper, based on a review of the literature, updates and summarizes the current knowledge about CX3CL1/CX3CR1 axis involvement in TBI and SCI pathogenesis, indicating possible molecular and cellular mechanisms with a potential target for therapeutic intervention.

## Introduction

Traumatic central nervous system (CNS) injuries are the most heterogeneous as regards the individual response to injury and the results of treatment of the group of diseases. In spite of constant progress concerning the pathophysiology, diagnostic work-up, and treatment, they still pose a challenge for the modern society due to their complex and prolonged course, socioeconomic dimension, and also numerous existing controversies concerning the implementation of optimal operative and non-operative treatment [1–4]. CNS injuries may be divided into traumatic brain injury (TBI) and spinal cord injury (SCI), depending on the anatomical location. Neurotrauma is a name which comprises both these types [5, 6]. The analysis of currently available data concerning long-term epidemiological research revealed that CNS injuries are one of the leading causes of death and severe disability which drastically reduces health-related quality of life (HRQoL) in all age groups and all populations all over the world, especially in the developing countries [7, 8]. According to the Center for Disease Control and Prevention (CDC), the epidemiological data on TBI for the years 2002–2006 shows the average of 1.7 million new cases of TBI in the USA, resulting in over 52,000 deaths and 80,000 cases of long-term disability [9–11]. According to 23 independent studies, the yearly incidence of TBI is estimated at 235 per 100,000 people in Europe [1, 12]. World Health Organization (WHO) claims that globally, TBI will surpass many diseases as the major cause of death and disability by the year 2020 [13]. Epidemiological data on SCI, based on statistical studies provided by National Spinal Cord Injury Statistical Center (NSCISC), shows the annual incidence of SCI in the USA to be approximately 12,500–20,000 new cases and currently 240,000–337,000 living survivors [14, 15]. In Europe, the incidence of SCI is estimated at 15–16 per one million people [16, 17]. The global incidence rate is estimated at approximately 23 SCI cases per one million which equals to almost 180,000 new cases per annum [17, 18]. Apart from isolated TBI or SCI which occur quite rarely, it is also possible that these two conditions are concomitant and also accompanied by injuries to other body parts (extracranial or extraspinal injuries) which then means a multiple trauma in a patient [19–24]. CNS trauma and associated extracranial and extraspinal injuries give an image of a debilitating condition, which does not only create physical and emotional costs for individuals. It is also a significant financial burden for the whole society [25, 26]. With an increasing amount of knowledge about the pathogenesis of changes during TBI and SCI, it becomes clear that the natural course of these diseases does not only cover the direct moment of injury but, more importantly, long-term neurodegenerative processes [27, 28]. TBI- and SCI-related pathologies occur as a result of two subsequent complex mechanisms which are not clearly demarcated: primary injury and secondary (delayed) injury [1–3]. Brain and spinal cord injuries develop as a result of an external mechanical force, whose character, intensity, direction, and duration determine the severity of the injury. This mechanism is categorized as a primary injury [1–3]. Therefore, as regards primary injury, we can distinguish blunt TBI/SCI resulting from an external mechanical force and a rapid acceleration/deceleration, penetrating TBI/SCI which occurs by damaging the continuity of neural tissue by a ballistic object, and blast TBI/SCI resulting from different shock waves, e.g., acoustic, electromagnetic, light, and thermal waves or their combination, which are responsible for diffuse function disorders and neural tissue destruction [1–4, 29, 30]. The macrostructural image of primary injury includes contusion and edema of the neural tissue, discontinuation of meninges, concomitant fractures and dislocations of cranial and spinal bones, injuries and dislocations of ligamentous structures, and the development of intra- and extra-axial hemorrhages both in the brain and in the spinal cord [1, 31–33]. The effect of the primary injury is additionally strengthened by the dislocation and compression of edematous neural tissue by damaged osseous and ligamentous structures, hematomas, and also by possible compression of cerebrospinal fluid (CSF) cisterns which significantly influences the increase in intracranial pressure (ICP) and intraspinal pressure (ISP) [32–37]. Microscopically, the direct effects of primary injury include the immediate death of cells resulting from the direct mechanical force and secondary compression, the disruption of vascular regulation, hypoperfusion and hypoxia of injured tissues, the dysfunction of neurovascular units forming blood-brain and blood-spinal cord barriers (BBB and BSCB), microporation and the disorders of cell membrane permeability, changes in the ionic composition of intracellular and extracellular space, rapid release of neurotransmitters from damaged cells, and the possibility of diffuse axonal injury (DAI) [38, 39]. The essence of the multidimensional character of molecular and biochemical events occurring during secondary injury is the specific continuation of the processes initiated by primary injury, which contributes to the image of numerous synergistic neurodegenerative mechanisms [40–42]. The mechanisms of secondary injury include progressive disorders in the vascular regulation, progressive disorders of cell membrane permeability, disorders of energy homeostasis associated with the dysfunction of the synthesis of mitochondrial adenosine triphosphate (ATP), the production of free radicals and lipid peroxidation, glutamate (Glu) excitotoxicity, calcium (Ca^2+^)-mediated neurotoxicity, programmed cell death (apoptosis), and local and systemic inflammatory response related to the inflow of immune cells and secretion of inflammatory mediators [1, 3, 41, 42]. In case of an injury, all the abovementioned and other numerous mechanisms at the cellular and subcellular level lead to time-dependent neurochemical dysregulation, demyelination, loss of neurons (NeuN^+^), and the development of a glial scar resulting in the dysfunction of the injured neural tissue in the brain and the spinal cord [43–45]. Both the local and systemic body response constitute an essential element of CNS injuries [46, 47]. Immune system response influences all organs, not only the injured ones (the brain and the spinal cord), via marked changes encompassing gene expression changes, the recruitment of a broad spectrum of cells, and secretion of inflammatory mediators [46–48]. Neuroinflammation is considered to be one of the leading elements of secondary injury [49]. The role of immune response in the pathogenesis of TBI and SCI has been regarded controversial, due to its possible positive and negative consequences [49, 50]. In the past few years, independent authors who have been trying to fully elucidate the pathogenesis of TBI and SCI gave more attention to phenomena occurring during the secondary injury, particularly those connected with the immunological component [51]. Ongoing research most commonly concentrates on the mechanisms connected with the inflammatory mediators (e.g., cytokines, including chemokines) secreted by cells located residually in the brain and the spinal cord and also by infiltrating CNS structures via blood vessels [52, 53]. It seems that the most promising therapeutic direction is the modulation of the inflammatory response by limiting its neurotoxic effect, enhancing the neuroprotective properties and promoting the regeneration of injured neural tissue [54, 55]. As regards the wide range of inflammatory mediators connected with CNS functions, chemokines have been attributed an increasing role both in physiological and pathological conditions in the past few years [56]. The knowledge about the role of chemokines in CNS functioning is so wide, that researchers distinguished a new class of neurotransmitters and neuromodulators and named them neurochemokines [57]. The multidirectional actions of neurochemokines include the participation in the embryogenesis of the nervous system, modulation of synaptic conductance, plasticity, and also their function in the pathogenesis of neurodegenerative disorders [57]. Researchers have described almost 50 chemokine ligands, out of which chemokine CX3CL1 (fractalkine) and its receptor CX3CR1 deserve special attention. Apart from properties which are typical for the remaining compounds in this group, chemokine has a different molecular structure and may function not only as a chemoattractant but also as an adhesive molecule. The increasing role of the CX3CL1/CX3CR1 axis has been noted in the physiological communication between neurons and microglial cells. Moreover, there are numerous newly discovered phenomena as regards the influence of the CX3CL1/CX3CR1 axis on CNS physiology and pathology, such as the effect on the synaptic plasticity, maturation, and activity and a marked effect on the functioning of hippocampal formation [58]. Up to now, the role of CX3CL1/CX3CR1 axis has not been widely discussed in the context of its presence and possible functions in the pathophysiology of TBI- and SCI-related phenomena. Therefore, it seems justified to describe and summarize the participation of CX3CL1 and its CX3CR1 receptor in the course of CNS injuries basing on the available professional literature. In order to present a comprehensive overview of the problem, we will perform a thorough analysis of the structure and functions of the CX3CL1/CX3CR1 axis, present its role in the physiological processes related to CNS functioning, and then, we will discuss its possible role in the course of CNS injuries with particular attention paid to each stage of the pathology and its subsequent consequences and also possible clinical implications.

## Molecular Organization and Biological Functioning of CX3CL1 and Its Dedicated Receptor CX3CR1

### CX3CL1—a Unique Two-Form Ligand Member of the CX3C (Delta) Subfamily

**Fig. 1:**
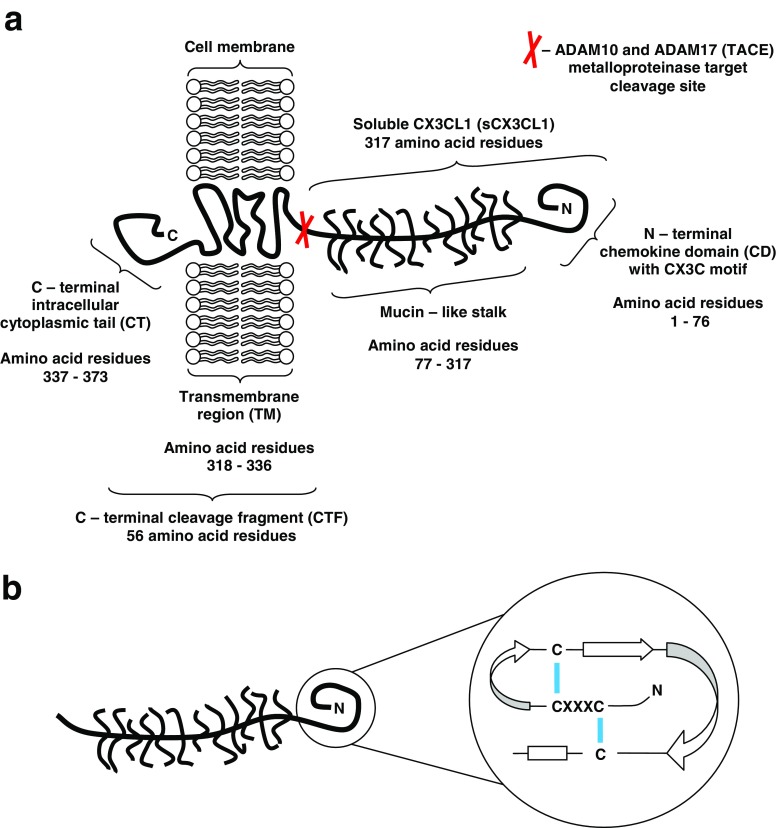
The schematic structure of two-form chemokine ligand CX3CL1 (fractalkine). **a** Membrane-anchored form of CX3CL1 showing specific regions of the molecule and the site of the cleaving action of the metalloproteinase ADAM10 and ADAM17 (TACE). **b** The soluble form of CX3CL1 (sCX3CL1), produced by metalloproteinase cleaving. The N-terminal chemokine domain (CD) containing the CX3C motif is shown in greater detail including important parts of the secondary and tertiary protein structure. Adopted and modified with permission from Wojdasiewicz P, Poniatowski ŁA et al. (2014) The Chemokine CX3CL1 (Fractalkine) and its Receptor CX3CR1: Occurrence and Potential Role in Osteoarthritis. Arch Immunol Ther Exp (Warsz) 62(5):395-403. doi: 10.1007/s00005-014-0275-0

In 1997, Bazan et al. first identified and described the CX3C (delta) subfamily of chemokines [59]. The name “fractalkine” was then used for the first time. In the same year (at the interval of several weeks), Pan et al. confirmed the existence of the CX3C subfamily [60]. This group of researchers used the name “neurotactin” to emphasize its mode of action during experimental autoimmune encephalomyelitis (EAE) on a mouse model. Regarding the latest classification of chemokines including the arrangement of amino acids and cysteine (Cys) residues with disulfide bonds, CX3CL1 is the only representative of the CX3C subfamily which is also classified as a dual-function chemokine (category D) which has the properties of homeostatic chemokines (category H) and inflammatory chemokines (category I) [61, 62]. In human genome, the gene coding CX3CL1 is composed of three exons and is located within the long arm of chromosome 16 (16q13) [63]. It is highly conservative with the genes of a mouse (*Mus musculus*) or a rat (*Rattus norvegicus*). The respective genes are located on chromosome 8 (8qC5) in the mouse and chromosome 19 (19p12) in the rat [64, 65]. In the body, CX3CL1 occurs in two different isoforms: membrane-anchored CX3CL1 and soluble CX3CL1 (sCX3CL1). Therefore, it has chemotactic and adhesive properties [66]. Ultrastructurally, the precursor form of membrane-anchored CX3CL1 is a polypeptide chain composed of 397 amino acid residues containing signal peptide (SP) included in 24 amino acid residues, N-terminal chemokine domain (CD) with CX3C motif included in 76 amino acid residues, forming a globular structure which is approximately 3 nm long, mucin-like stalk included in 241 amino acid residues which are 26 nm long, also including 17 degenerated mucin-like repeats with 26 potential O-glycosylations at serine (Ser) and threonine (Thr) residues, transmembrane region (TM) included in 21 amino acid residues, and C-terminal intracellular cytoplasmic tail (CT) included in 35 amino acid residues [59, 60, 67, 68] (Fig. 1). The mature membrane-anchored form of CX3CL1 does not contain SP and is composed of 373 amino acid residues of the total molecular weight of approximately 17.5 kDa, and 95 kDa after glycosylation [59, 60]. The connection with cell membranes is a factor which, apart from the regulation at the level of transcription and translation observed in other chemokines, is associated with an additional phenomenon of functional regulation via undergoing constitutive internalization, producing a dynamic balance in membrane compartments between the plasma membrane and the intracellular endocytic compartment [69–71]. The structure of intracellular CT includes two adaptor protein 2 (AP2)-binding motifs, such as YQSL at positions 362–365 and YVLV at positions 392–395 (PSORT II analysis), predicted to bind clathrin-coated pit which allows constitutive clathrin-mediated endocytosis [70]. The distribution of CX3CL1 in distinct subcellular compartments is also associated with the properties of soluble N-ethylmaleimide-sensitive factor attachment protein receptor (SNARE), such as syntaxin 13 (STX13) and vesicle-associated membrane protein 3 (VAMP3) [71]. This type of dynamic balance facilitates the protection of presynthesized CX3CL1 against premature degradation on the external side of cell membrane and, under appropriate conditions, accelerates the mobility of intracellular content [70, 71]. Another form to be described is sCX3CL1 included in 317 amino acid residues, approximately 29 nm long, whose structure encompasses a CD-containing segment and a mucin-like stalk of the total molecular weight of approximately 14.7 kDa, and 80 kDa after glycosylation [59, 60]. The separation of sCX3CL1 results from proteolytic cleavage process which occurs at the di-arginine (di-Arg) motif (RR) near the external surface of plasma membrane [59]. The process is based on ectodomain shedding in which sCX3CL1 acts as a soluble ectodomain and is mediated by two transmembrane proteases from a disintegrin and metalloproteinase (ADAM) family and by cathepsin S (CTSS) [72, 73]. The proteases from ADAM family, which take part in this process are ADAM metallopeptidase domain 10 (ADAM10) and ADAM metallopeptidase domain 17 (ADAM17) which is also called tumor necrosis factor-alpha-converting enzyme (TACE) [72]. ADAM10 was identified as a protease responsible for the constitutive and ionomycin-induced shedding of CX3CL1, and ADAM17 is involved in the 12-O-tetradecanoylphorbol-13-acetate (PMA)-induced shedding of CX3CL1 [74, 75]. It is worth noting that the described shedding events may be pharmacologically targeted by the use of a specific inhibitor for ADAM10 (GI254023X) and the dual-specific ADAM10 and ADAM17 inhibitor (GW280264X) [74, 76]. The remaining C-terminal cleavage fragment (CTF) undergoes degradation associated with the activity of membrane-associated secretase complexes and the proteasome [77].

### Characteristics of CX3CR1 Chemokine Receptor

**Fig. 2:**
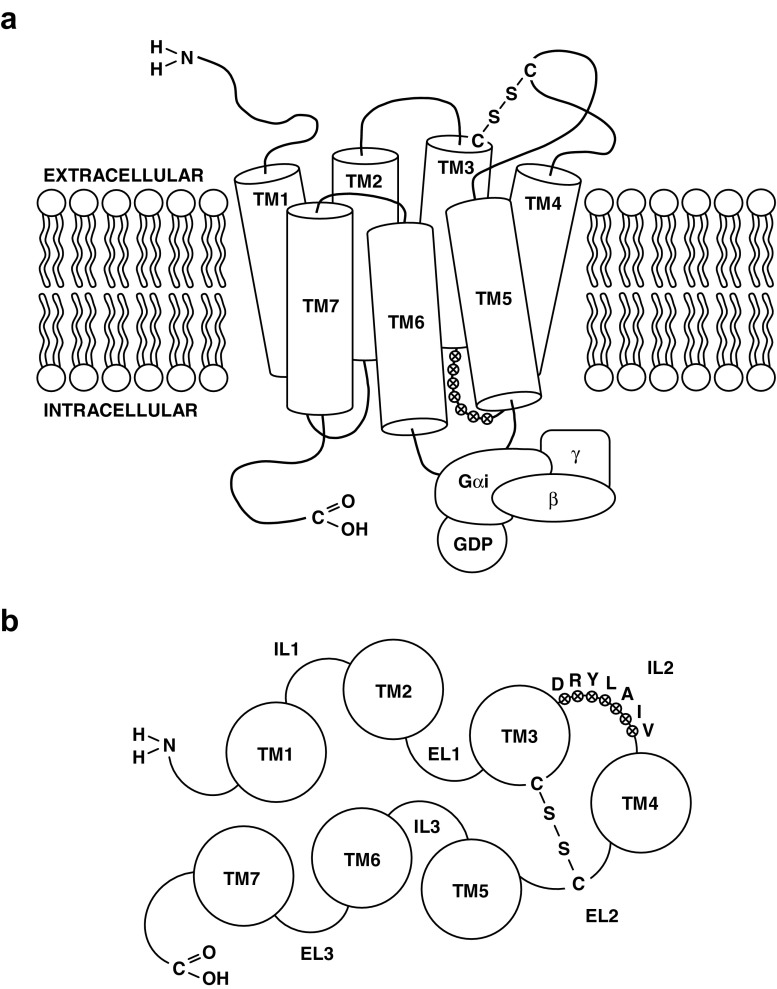
The schematic structure of CX3CR1 chemokine receptor. The molecular structure of the receptor includes seven α-helical transmembrane domains (TM1–TM7), three extracellular (EL1, EL2, EL3) and three intracellular loops (IL1, IL2, IL3), an extracellular N-terminus, and an intracellular C-terminus. The *disulphide bond* is shown between two highly conserved cysteines (Cys) which are located respectively at the top of the third transmembrane domain (TM3) and the second extracellular loop (EL2). The second intracellular loop (IL2) contains a conserved seven amino acid sequence Asp-Arg-Tyr-Leu-Ala-Ile-Val (DRYLAIV motif) which serves as Gαi heterotrimeric protein docking site. **a** CX3CR1 receptor shown from the side perspective. **b** CX3CR1 receptor shown from the intracellular perspective

Unlike most ligands belonging to the chemokine family with the affinity for numerous receptors, CX3CL1 reveals its biological activity via an interaction with only one dedicated receptor—CX3CR1 [78]. During a study on a rat model conducted by Harrison et al. in 1994, CX3CR1 was first described as RBS11 constituting the orphan G protein-coupled receptor (oGPCR) [79]. In further study by Raport et al., a homologous human gene was named V28, and its product was described as oGPCR containing a highly conserved 20 amino acid region typical for receptors from G protein-coupled receptor (GPCR) family and seven amino acid sequence Asp-Arg-Tyr-Leu-Ala-Ile-Val (DRYLAIV motif) in the second cytoplasmic loop (IL2) which is conserved among chemokine receptors [80]. The identification of the connection of CX3CL1 and CX3CR1 in a signaling axis was described by Imai et al. in 1997 [81]. In human genome, the gene coding CX3CR1 is composed of four exons and is located within the short arm of chromosome 3 (3p21.3) [82, 83]. It is conservative with mouse or rat genes, which, in turn, are located on chromosome 9 (9qF4) in the mouse and on chromosome 8 (8q32) in the rat [80, 82–84]. According to existing classifications, CX3CR1 belongs to the biggest A class (rhodopsin-like receptors) of GPCR proteins, which includes all chemokine receptors [85, 86]. Ultrastructurally, the polypeptide chain of CX3CR1 includes 355 amino acid residues of the total molecular weight of approximately 40 kDa [80] (Fig. 2). The polypeptide chain includes extracellular N-terminus, seven α-helical domains (TM1–TM7) penetrating the whole thickness of the cell membrane, three intracellular (IL1, IL2, IL3) and three extracellular loops (EL1, EL2, EL3), and an intracellular C-terminus [87]. Two conservative Cys residues located respectively at the top of the third transmembrane domain (TM3) and the second extracellular loop (EL2) are connected with a disulfide bond [88]. N-terminus and loops formed by the polypeptide chain extracellularly create the location of binding two functional ligands, which can be both CX3CL1 and CCL26 (eotaxin-3) and also antibodies and some pathogens such as bacteria and viruses [89–92]. It was also demonstrated that the appropriate level of tyrosine (Tyr) sulfation of N-terminus is necessary for maintaining normal activity of the majority of GPCR receptors for chemokines [93]. C-terminus and loops located on the cytoplasmatic side form the site where heterotrimeric Gαi protein is bound [81]. The phosphorylation of C-terminus, which occurs easily due to numerous Ser and Thr residues, facilitates the appropriate modulation of the process of transmitting a signal associated with Gαi protein [94]. Gαi protein, which has a function in the transmission of intracellular signals associated with CX3CR1 activation, may be inhibited by pertussis toxin (PTX), which was implemented in some experimental models [90]. The majority of chemokine GPCR receptors (including CX3CR1) are characterized by a marked polymorphism. This may be responsible for intraindividual and interindividual variability of chemokine effects and may account for changeable risk of the development and course of inflammatory, autoimmune, and hyperplastic diseases [95, 96]. It is also worth noting that the existence of only one receptor for CX3CL1 significantly facilitates the interpretation and possible clinical implications of reported biological effects concerning the CX3CL1/CX3CR1 axis.

### Systemic Expression and Distribution of the CX3CL1/CX3CR1 Axis

The main source of chemokines is white blood cells (WBC), but CX3CL1 is mainly produced in endothelial cells and neurons [97]. It is not surprising that CX3CL1 expression is elevated in highly vascularized and well-innervated organs and also in locations with an increased concentration of immune system cells, such as the CNS, lungs, cardiac muscle, liver, intestines, and placenta [98–104]. In spite of the wide distribution and expression throughout the body, CX3CL1 presents a high specificity for some cell types. CX3CR1^+^ cells present chemotactic properties toward sCX3CL1. They include a significant percentage of immune cell population, i.e., monocytes (CD14^+^), macrophages (MΦ), NK cells (CD16^+^), lymphocytes (CD4^+^ and CD8^+^), mast cells (MC), and dendritic cells (DC) [105–109].

### CX3CL1/CX3CR1 Axis in the Regulation of Cellular Mechanism Involved in Cellular Adhesion and Migration

Leukocyte transendothelial migration (TEM) from vascular lumen into extravascular tissues triggers a dynamic cascade of molecular events connected with interactions between leukocytes and endothelial cells both under physiological and pathological conditions [110]. Apart from the conventional leukocyte extravasation based on integrin-mediated adhesion, it is also possible to employ the adhesive activity of membrane-anchored CX3CL1 [111]. It was noted that the CX3CL1/CX3CR1 axis is involved at all migration stages, but the adhesive ability of CX3CL1 is determined by the presence of mucin-like stalk [111, 112]. Leukocytes which have an appropriate level of CX3CR1 expression adheres CD to membrane-anchored CX3CL1 [112]. Following the adhesion associated with an interaction between CX3CL1 and CX3CR1, leukocytes are able to migrate through vessel walls in a directly selectin- and integrin-independent manner [113]. Moreover, after the adhesion of CX3CL1 to CX3CR1, there is an activation and synthesis of other adhesive molecules which strengthens the adhesion via synergism [114, 115]. It was also noted that integrins may act as receptors and co-receptors as they are able to adhere to CX3CL1 with no participation of CX3CR1 [114, 115]. Multilevel interactions of the CX3CL1/CX3CR1 axis with different types of cell adhesion molecules (CAM), such as selectins and integrins, are currently thought to be key phenomena in the pathogenesis of many inflammatory diseases, which may be useful as potential locations of modulating immune response associated with the recruitment of individual cell types [116].

### CX3CL1/CX3CR1 Axis Interplay with Related Activators/Repressors and Intracellular Multiple Signaling Pathways

The regulation of the activation and functioning of the CX3CL1/CX3CR1 axis encompasses the control at the level of transcription and also regulation during post-translational modifications. Intracellular transmission of signals via CX3CR1 and then heterotrimeric Gαi protein is associated with the activation of numerous signaling molecules, such as several different secondary messengers and transcription factors, including nuclear factor kappa-light-chain-enhancer of activated B cells (NF-κB), cAMP response element-binding protein (CREB), signal transducer and activator of transcription (STAT), and activator protein 1 (AP-1) [117–119]. The activation of the transcription factors associated with the CX3CL1/CX3CR1 axis may be connected with activating a potentially wide range of functions of a given cell, such as messenger RNA (mRNA) transcription for many proteins (including cytokines), cytoskeletal rearrangement and migration, apoptosis, and proliferation [117, 119]. Local production and membrane expression of CX3CL1 and also CX3CR1 are controlled by other cytokines with the most important ones including tumor necrosis factor alpha (TNFα), interleukin 1 (IL-1), interferon gamma (IFNγ), and soluble interleukin 6 receptor alpha (sIL-6Rα) [120, 121]. Similar reciprocal correlations were also observed as regards the presence of lipopolysaccharide (LPS), nitric oxide (NO), adenosine (ADO), 15-deoxy-delta(12,14)-prostaglandin J(2) (15d-PGJ(2)), 8-isoprostane, and hypoxia [122–126]. CX3CL1 production induced by various factors is also subjected to autoregulation via modulating the expression of their CX3CR1 receptor [117]. The activation and functioning of individual cascades of intracellular pathways associated with the CX3CL1/CX3CR1 axis under particular physiological and pathological conditions demonstrate differences, and a direct recapitulation of their activity in a specific clinical status within various cell and tissue types requires further research which would confirm these potential correlations.

## Overview of the Role of CX3CL1 and CX3CR1 in the Physiological Functioning of the Brain and Spinal Cord

### CX3CL1/CX3CR1 Axis—Neuroanatomical Ultrastructural Location and Regional Distribution

The analysis of in vitro experiments performed on cellular models and knowledge about transgenic mouse and rat models facilitates deep insight into the pattern of CX3CL1 and CX3CR1 expression within the CNS. In order to comprise the extensive neuroanatomical distribution of CX3CL1 and CX3CR1, it needs to be noted that high levels of CX3CL1 chemokine are constitutively produced by neurons within the telencephalon and diencephalon and particularly in the cerebral cortex, hippocampus, amygdala, basal ganglia, thalamus, and olfactory bulb [127–129]. A particularly high level of expression of CX3CL1 mRNA transcripts is present in the hippocampal formation demonstrating the highest concentration in CA1, CA2, and CA3 regions and in the cerebral cortex—layers II, III, V, and VI [127–129]. The hypothalamus and mesencephalon are the areas where the levels of mRNA expression for CX3CL1 are very low or even below detectable limits [127–129]. A similarly low expression level was noted within the metencephalon and myelencephalon encompassing the pons, cerebellum, and medulla oblongata [127–129]. Interestingly, mRNA expression for CX3CL1 is mainly associated with expression within the gray matter and the lack of its expression within the white matter, e.g., within the corpus callosum and fimbria/fornix (FF) structures [129, 130]. CX3CL1 expression within the spinal cord is limited to neurons in the dorsal horn and dorsal root ganglia (DRG) [131, 132]. Interestingly, it remains controversial that endothelial cells in the brain and the spinal cord, as opposed to those in other locations, do not present constitutive CX3CL1 expression on the surface, which suggests that it is rather dependent on the activation occurring as a result of an inflammation in certain CNS pathologies [127, 129, 133, 134]. Constitutive mRNA expression for CX3CL1 and CX3CR1 is also described by astrocyte cells (GFAP^+^) [135, 136]. In the ventricular system, CX3CL1 and CX3CR1 expression is connected with the choroid plexus (CP) [137, 138]. CX3CL1 occurs physiologically in the CSF at the concentration which is approximately 500-fold below serum concentration [139]. It is widely accepted that CX3CR1 demonstrates a relatively homogeneous expression via microglial cells in the brain and the spinal cord which, at the same time, do not demonstrate mRNA expression for CX3CL1 [127–129, 135]. Apart from the abundant presence of CX3CR1 receptor within microglial cells it is also important to note its expression via neurons [140–142]. When analysing the presence of CX3CR1 both on neurons and on microglial cells, it is important to note the possibility of distinguishing between the direct modulating effect of CX3CL1 on neurons and its indirect effect via previously activated microglial cells [142, 143]. Making such a distinction is possible by the analysis of the effect of CX3CR1 activation on glutamatergic transmission which results in the inhibition of phosphorylation of α-amino-3-hydroxy-5-methyl-4-isoxazolepropionic acid (AMPA) receptor and, more precisely, its glutamate receptor 1 (GluR1) subunit associated with calcium oscillator, increased Ca^2+^ entry, and the reduction of excitatory postsynaptic current (EPSC) amplitude [144, 145]. It was also noted that CX3CL1 inhibits hippocampal long-term potentiation (LTP) in CA1 region through adenosine A3 receptor (A3R) activity [146]. Differences in mRNA expression for CX3CL1 and CX3CR1 are also correlated with age, where the reduction in CX3CL1 expression and level and the increase in CX3CR1 level within the hippocampus are accompanied by an increase in microglial activity through the phosphorylation of protein kinase B (Akt) and activation of the phosphatidylinositol-4,5-bisphosphate 3-kinase (PI3-K) pathway [147].

### CX3CL1/CX3CR1 Axis in the Context of Homeostatic Bidirectional Cross Talk Between Neurons and Microglia

**Fig. 3:**
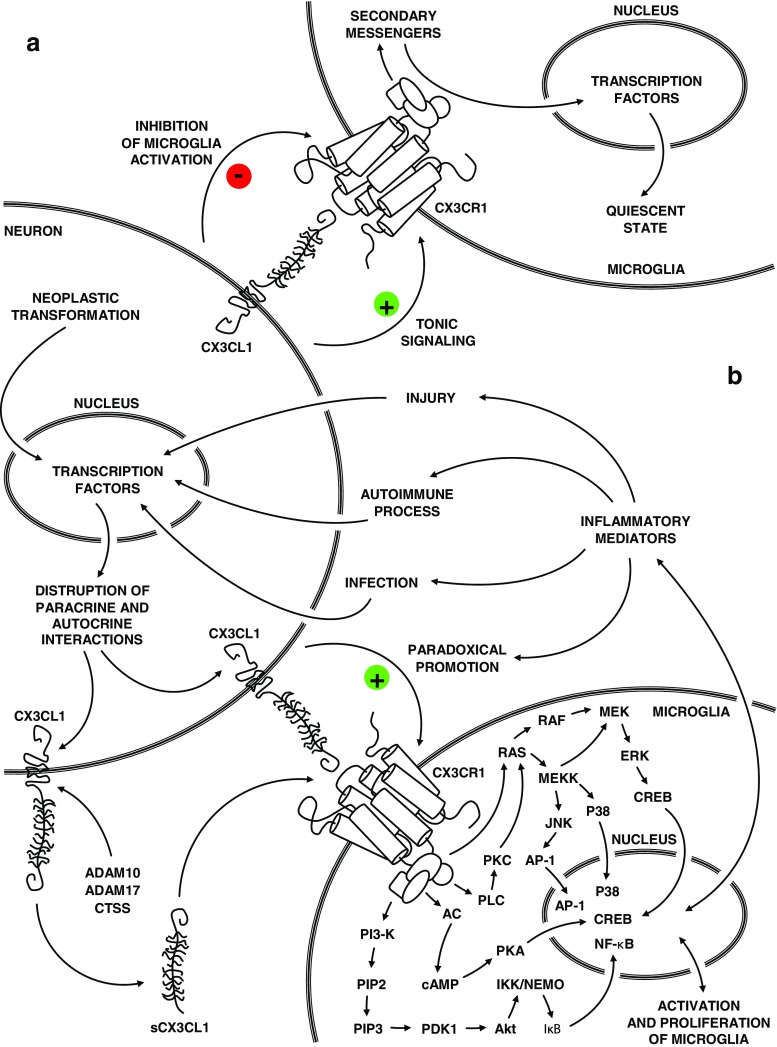
The schematic representation of the physiological and pathological role of CX3CL1/CX3CR1 axis in the context of bidirectional cross talk between neurons and microglia. **a** Under physiological conditions, microglia undergo tonic signaling through the CX3CL1/CX3CR1 axis, which facilitates the maintenance of its cells in a quiescent state. **b** Under pathological conditions, the homeostasis cross talk between neurons and microglia through the CX3CL1/CX3CR1 axis is disrupted. Upregulated levels of several cytokines, chemokines, and other mediators create a specific inflammatory environment, which results in paradoxical promotion through the CX3CL1/CX3CR1 axis, activation and proliferation of microglia, and the infiltration of peripheral immune cells

Regarding both the neuroanatomical CX3CL1 and CX3CR1 distribution in the context of bidirectional interaction between neurons and microglial cells, it can be noted that the CX3CL1/CX3CR1 signaling axis probably has a crucial role in modulating the homeostasis of these multilevel interactions throughout the ontogenetic development of mammals [148]. Microglia, which seem to be constantly active, undergo tonic signaling through the CX3CL1/CX3CR1 axis under physiological conditions, which facilitate the maintenance of its cells in a quiescent state and maintain homeostasis in the neuronal network [148, 149]. The phenomenon seems to corroborate the fact that structural connectivity reflects functional connectivity, particularly influencing synaptic transmission in specific neuroanatomical areas, in processes like learning, memory, and behavior [58, 150]. However, the unambiguous determination of the role of the CX3CL1/CX3CR1 axis as inflammatory or anti-inflammatory seems controversial in the context of functioning within the CNS [148]. Microglia, which is the non-neuronal element of the CNS, includes specific residual macrophages performing the function of a specific sensor sensitive to injuries and developing pathologies in the neural tissue, e.g., an injury or a neoplastic, autoimmune, or infectious process [151, 152]. Under physiological conditions, CX3CL1 seems to inhibit microglial activation, while in particular pathologies a paradoxical promotion of inflammatory response may occur [151, 153, 154]. The factors which determine the specific type of neuroprotective or neurotoxic response are most probably dependent on the type of the primary destructive factor, CNS area, and the local concentration of CX3CL1 and CX3CR1 [148]. The disruption of homeostatic paracrine and autocrine interactions of the CX3CL1/CX3CR1 axis in the context of neuron-microglia communication may be seen as one of fundamental elements in the pathogenesis of CNS-related diseases [155] (Fig. 3). The understanding and determination of the precise location of the CX3CL1/CX3CR1 signaling axis as a physiological element of cell properties within the CNS still require further detailed research.

## Role of CX3CL1 and CX3CR1 Axis in the Pathophysiology of Traumatic Brain and Spinal Cord Injury

### Expression Pattern of the CX3CL1 and CX3CR1

The properties of CX3CL1 and CX3CR1 and also of the remaining chemokines and their receptors are still the focus of research on humans and various animal models, to present their potential role and possibility of implementation in the clinical therapy of TBI and SCI. The analysis of data available in the professional literature demonstrates the scarcity of studies conducted in patients and the domination of animal model and cell culture approach.

#### Traumatic Brain Injury

Available studies concerning the level of CX3CL1 and CX3CR1 expression in the course of TBI present data including both clinical trials with patients suffering from severe head injuries and also data obtained from animal model and cell culture studies (Table 1). Rancan et al., who conducted a study in patients with head trauma and in mice after standardized cortical experimental contusion, were the first to analyze the posttraumatic profile of CX3CL1/CX3CR1 axis expression in TBI [156]. The study group included 12 patients (*n* = 12) with Glasgow Coma Scale (GCS) of ≤8 on admission and with visible changes in computed tomography scans according to Marshall classification [157]. The patients had intraventricular catheters (IVCs) for the direct measurement of ICP. CSF drainage was performed when the pressure was over 15 mm Hg. The control group for CSF analysis included five patients (*n* = 5) without neuropathologies, and as regards serum analysis, there were eight (*n* = 8) healthy volunteers. The levels of CX3CL1 in CSF obtained in the control group were 12.6 to 57.3 pg/mL, while in the study group, they were 29.92 to 535.33 pg/mL demonstrating the most abundant values on the day of admission, with a gradual decreasing tendency during 14 days of the study. CX3CL1 levels in the serum of the control group patients were 21.288 to 74.548 pg/mL, while in the study group, they amounted to 3.1 to 59.159 pg/mL. Therefore, the authors reported that physiologically, the concentration of CX3CL1 in humans is higher in the serum than in the CSF and the values are reversed as a result of TBI, correlating with BBB dysfunction and suggesting the possibility of shifting CX3CL1 from the serum to the CSF. The second part of the study was performed on a mouse model (*n* = 40). No differences were observed as regards CX3CL1 levels and the expression of its mRNA in the study group and sham-operated control group. However, the authors noted an elevated level of mRNA for CX3CR1 in the study group which gradually increased between the trauma and the seventh day of the experiment. Helmy et al. conducted a study which consisted in the determination of cytokine profile in patients with TBI [158]. They performed cerebral microdialysis (CM) with two types of perfusates, such as crystalloid perfusate and 3.5 % human albumin solution (HAS) and the serum coming from sampled arterial and venous blood. The qualified patients (*n* = 12) had the GCS of ≤8 on admission and typical changes in the computed tomography scan. With crystalloid perfusate, the median CX3CL1 concentration was 9.21 pg/mL, and with 3.5 % HAS, it was 20.39 pg/mL. The median microdialysate concentration/arterial plasma ratio was 0.96. Gaetani et al. performed an analysis of human brain samples harvested during decompressive craniotomy for TBI and after spontaneous intracranial hemorrhage (ICH) [159]. They were studied in terms of CX3CL1 and CX3CR1 expression. The level of CX3CL1 expression demonstrated upregulation in the neural compartment compared to samples from the control group harvested during gyrectomy, which was a part of a surgery for unruptured intracranial aneurysyms (UIAs). It was demonstrated that the level of CX3CL1 expression correlated with lower ICP within the glial compartment. CX3CR1 expression was observed in lower concentrations both in neurons and glia, while its higher concentrations in neurons were analyzed with regard to the GCS of patients on admission. Fahlenkamp et al. analyzed organotypic hippocampal slice cultures in a mouse model [160]. The cultures were subjected to in vitro mechanical dropweight trauma of moderate severity in the CA1 region to assess local cytokine and chemokine reaction. The results demonstrated the downregulation of the expression of transcript mRNA for CX3CL1 with simultaneous upregulation of the expression of transcript mRNA for CX3CR1.

**Table 1:** Observational studies showing the expression pattern of CX3CL1 and CX3CR1 in traumatic brain injury

Reference	Clinical trial/model	Method of sampling	Methodology for quantification	Reported results/timing			
				CX3CL1	CX3CR1	CX3CL1 mRNA	CX3CR1 mRNA
Rancan et al. [156]	Patients with TBI	CSF intraventricular draining catheters, blood samples	ELISA	Increase of concentration in CSF, remains increased at 14 days			
				Decrease of concentration in the serum, remains decreased at 14 days			
				Correlation of concentration in CSF with BBB dysfunction			
	Mouse with TBI	Brain samples	ELISA, Northern blot	No difference in concentration		No difference in expression	Increase in the expression, remains increased at 7 days
Helmy et al. [158]	Patients with TBI	Cerebral microdialysis, blood samples	ELISA	Median concentration 9.21 or 20.39 pg/mL dependent of perfusate type			
				Median extracellular/arterial plasma ratio 0.96			
Gaetani et al. [159]	Patients with TBI or spontaneous ICH	Brain samples	IHC	Increase of concentration in the neural compartment	Correlation of concentration with GCS		
				Correlation of concentration with ICP			
Fahlenkamp et al. [160]	Drop-weight trauma in the CA1 region of the mouse hippocampus	Organotypic cell slice cultures	RT-PCR			Decrease of expression	Increase of expression

*TBI* traumatic brain injury, *ICH* intracranial hemorrhage, *CA1* cornu ammonis 1, *CSF* cerebrospinal fluid, *ELISA* enzyme-linked immunosorbent assay, *IHC* immunohistochemistry, *RT-PCR* real-time polymerase chain reaction, *BBB* blood-brain barrier, *ICP* intracranial pressure, *GCS* Glasgow Coma Scale

#### Spinal Cord Injury

The analysis of CX3CL1 and CX3CR1 expression levels in the course of SCI has only been possible to perform with studies conducted on animal models (Table 2). Detloff et al. conducted a series of experiments concerning behavioral and cellular responses after thoracic (Th8) SCI with the use of electromagnetic impactor in rats, occurring below the level of injury in the lumbar spinal cord (L5) [161]. It was demonstrated that within L5 dorsal horn, the level of CX3CL1 presented no changes after 7, 21, and 35 days following the SCI, while the level of mRNA expression for CX3CL1 decreased after 35 days following the SCI. The level of mRNA expression for CX3CR1 was elevated compared to control groups. Donnelly et al. conducted a study on a mouse model with SCI in the thoracic spine (Th9–Th10) [162]. It was demonstrated that the local mRNA expression for CX3CL1 was decreased between days 1 and 7 after SCI, and it reached its normal level after 14 days. On day 28, it had a markedly elevated level. However, mRNA expression for CX3CR1 was reduced between days 1 and 3 after SCI. Then, the expression gradually increased between days 3 and 28 in comparison with control groups. The expression of CX3CL1 remained unchanged for 6 weeks after SCI, while CX3CR1 demonstrated an increased expression starting on the third day after SCI which corresponded with the level of expression of its mRNA. Cizkova et al. reported spatial changes in the expression of CX3CR1 within rostro-caudal axis after SCI on the rat model after 3 days, with the spinal cord damaged in the thoracic region (Th8–Th9) with the use of modified balloon compression technique (2-French Fogarty catheter) [163]. It was noted that the CX3CR1 expression significantly increased after 3 days in the study group (*n* = 4) in comparison with sham-operated control group (*n* = 4) where CX3CR1 expression was homogeneous and poorly pronounced. The expression was particularly intensified within the gray and white matter of the rostral segment (Th2–Th6) compared to the caudal segment (Th12–L3). Blomster et al. (2013) analyzed CX3CL1 concentrations in the serum of the blood sampled with cardiac puncture from the left ventricle (LV) in mice subjected to SCI in the thoracic spine (Th9) [164]. It was demonstrated that 7 days after, SCI mice (*n* = 6) presented a slightly elevated CX3CL1 concentration in comparison with a group of non-injured (*n* = 5) and sham-operated (*n* = 5) controls.

**Table 2:** Observational studies showing the expression pattern of CX3CL1 and CX3CR1 in spinal cord injury

Reference	Clinical trial/model	Method of sampling	Methodology for quantification	Reported results/timing			
				CX3CL1	CX3CR1	CX3CL1 mRNA	CX3CR1 mRNA
Detloff et al. [161]	Rat with SCI	Spinal cord samples	IHC, ELISA, RT-PCR	No difference in concentration after 35 days		Decrease in the expression after 35 days	Increase in the expression after 35 days
Donnelly et al. [162]	Mouse with SCI	Spinal cord samples	IHC, RT-PCR, Western blot	No difference in concentration	Increase in concentration, remains increased at 28 days	Decrease in the expression at 1–7 days and returns to baseline after 14 days	Decrease in the expression at 1–3 days and then remains increased at 3–28 days
Cizkova et al. [163]	Rat with SCI	Spinal cord samples	IHC		Increase in concentration especially in injured lesion and rostral segments to the lesion after 3 days		
Blomster et al. [164]	Mouse with SCI	Blood samples	ELISA	Slightly increase in concentration in the serum after 7 days			

*SCI* spinal cord injury, *IHC* immunohistochemistry, *ELISA* enzyme-linked immunosorbent assay, *RT-PCR* real-time polymerase chain reaction

### Implication on Systemic and Local Mechanisms Involved in the Course of Traumatic Brain and Spinal Cord Injury

Regarding the kinetics of the changes of CX3CL1 and CX3CR1 expression occurring as a result of TBI and SCI, it becomes obvious that the regulation within this signaling axis should be regarded as an endogenous mechanism of the control of an inflammation resulting from this type of injuries (Table 3). CNS trauma triggers the local activation and migration of microglia, enhances the secretion of inflammatory mediators like cytokines and chemokines, and creates a specific inflammatory environment, which also results in the infiltration of peripheral immune cells [165, 166]. As regards the possible destruction and regeneration of CNS tissues, the presence of activated macrophages seems to be of key importance. In spite of the fact that the macrophages are of different origin and, partially, phenotype, they are similar in numerous aspects [167–169]. The sources of the activated macrophages include both microglia and the population of monocytes mobilized from circulation originating from bone marrow and splenic reservoir [164, 169]. Both cell types demonstrate a similar morphology and the expression of similar superficial markers [170]. The analysis of a mouse model in terms of the superficial expression of receptor markers demonstrated that microglia are immunophenotypically described as Ly6C^low^/CX3CR1^high^/CD45^low^/Iba-1^+^, while circulating monocytes are described as two subpopulations, such as inflammatory monocytes (classically activated, M1) defined as Ly6C^high^/CX3CR1^low^/CCR2^high^ and anti-inflammatory monocytes (alternatively activated, M2) defined as Ly6C^low^/CX3CR1^high^/CCR2^low^ [171–175]. Human counterparts of Ly6C^high^ and Ly6C^low^ monocytes are defined as CD14^++/^CD16^−^ and CD14^+^/CD16^++^ [175, 176]. The main source of knowledge regarding the direct functioning of the CX3CL1/CX3CR1 axis in TBI and SCI is studies on animal models which were subjected to substitution on one or both CX3CR1-coding alleles using gene for the green fluorescent protein (GFP) in this way obtaining heterozygotes (CX3CR1^+/GFP^) or knockout (KO) homozygotes (CX3CR1^GFP/GFP^). If we analyze studies by Donnelly et al. and Blomster et al. (2013) at a different angle, we may note that the modulation of monocyte activation via changes in the CX3CL1/CX3CR1 signaling axis on chimeric mouse models with SCI may affect their course [162, 164]. In the study of Donnelly et al., it was observed that the bone marrow of CX3CR1^GFP/GFP^ chimeric mouse, contrary to wild-type (WT) mouse, presents a negative effect on the recruitment and maturation of macrophages defined as Ly6C^low^/iNOS^+^/MHCII^+^/CD11c^−^ at the site of injury along with their capacity of the production of inflammatory cytokines and oxidative metabolites, with a positive effect on the recruitment of macrophages defined as Ly6C^high^/CCR2^+^/MHCII^−^/CD11c^+^ [162]. Microglia activation was also altered, which was expressed as the reduced production of mRNA for interleukin 6 (IL-6) and inducible nitric oxide synthase (iNOS) with the absence of changes in the production of mRNA for interleukin 1 beta (IL-1β) and TNFα. Moreover, when using the standardized Basso Mouse Scale (BMS), CX3CR1^GFP/GFP^ mice demonstrated a faster and sustained resumption of mobility, unlike WT mice [177]. Blomster et al. (2013) performed a study on the bone marrow of CX3CR1^GFP/GFP^ mouse chimeras [164]. They noted that CX3CR1 deficiency contributed to an increased recruitment of monocytes with no effect on their distribution and content at the site of injury. The BMS of CX3CR1^GFP/GFP^ mice indicated the deteriorated recovery of locomotor function in comparison with WT mice. Another study conducted by Blomster et al. (2011) concerning olfactory bulbectomy (OBX) in CX3CR1^GFP/GFP^ mice showed some properties of the CX3CL1/CX3CR1 axis in the course of TBI, because a model injury of olfactory system revealed some aspects if the injury did not result from a direct impact, but from surgical intervention [178]. An increased recruitment of monocytes was observed in the olfactory neuroepithelium of CX3CR1^GFP/GFP^ mice in comparison with WT mice. The level of mRNA expression for ADAM10 was slightly increased, while no changes were noted in mRNA expression for ADAM17 which were independent from the genotypes of the mice in the study. Significant changes were noted as regards the expression of inflammatory cytokines, which was demonstrated as an increased expression of mRNA for IL-1β, IL-6, and TNFα in CX3CR1^GFP/GFP^ mice in comparison with WT mice. Morganti et al. studied TBI on a mouse model using CX3CR1^GFP/GFP^ chimeras [179]. They observed neurotoxic responses at acute (24 h) and chronic (3 months) stages after controlled cortical impact (CCI). It was noted that 24 h after an injury, the inflammatory response in CX3CR1^GFP/GFP^ mice was decreased which manifested as reduced IL-1β, IL-6, TNFα, and iNOS expression in comparison with WT mice. The analysis of hippocampal-dependent cognitive function 3 months following an injury revealed that CX3CR1^GFP/GFP^ mice made fewer mistakes in the radial arm water maze (RAWM) test than WT mice. As regards changes in synaptic conductance, it was demonstrated that a quantitative change of a NR2B subunit of N-methyl-D-aspartate (NMDA) receptor occurred within the hippocampus of a WT mouse, while it was not observed in CX3CR1^GFP/GFP^ mice. Other investigated differences in both types of mice include the effect on the activity of Src kinase, p44/42 MAP kinase, postsynaptic density protein 95 (PSD-95), and tau protein phosphorylation. Zanier et al. conducted a similar study on CX3CR1^−/−^ mouse model [180]. The mice were subjected to CCI, and a number of parameters were compared after 4 days and 5 weeks following the TBI. It was noted that 4 days after the injury, there were no changes in IL-1β and TNFα expression in CX3CR1^−/−^ mice in comparison with WT mice. However, there was a reduction in iNOS expression with simultaneous unchanged level of CD11b^+^ marker. During the fifth week, the expression of IL-1β and TNFα still remained unchanged, but the expression of iNOS and CD11b^+^ marker increased. Moreover, the levels of expression of interleukin 10 (IL-10) and insulin-like growth factor 1 (IGF-1) were also assessed. After 4 days and during the fifth week, their expression was lower in CX3CR1^−/−^ mice in comparison with WT mice, and their elevated levels were noted only 1 day after TBI. As regards the neurological outcome, CX3CR1^−/−^ mice demonstrated superior results on day 4 after TBI in comparison with WT mice in neuroscore test [181]. However, during week 5, their results were poorer. Regarding numerous parameters assessed in this study, it was stated that blocking CX3CL1/CX3CR1 axis conduction leads to positive effects soon after TBI, but the long-term outcome is poorer. Another valuable study with a similar methodology is one conducted by Febinger et al. on the mouse model of KO CX3CR1^GFP/GFP^ homozygotes which were subjected to CCI [182]. The mice were compared as regards the assessed parameters in two time intervals since the injury, both in the acute phase (24 h to 15 days) and in the chronic phase (15 to 30 days) with particular emphasis put on the assessment of locomotor activity, motor learning, anxiety behavior, and cognitive function with the following tests: neuroscore, open field, elevated plus maze (EPM), rota-rod, and Morris water maze (MWM). It was noted that in the acute phase, the CX3CR1^GFP/GFP^ chimeric mice demonstrated superior results in comparison with WT mice in neuroscore test, while in the chronic phase, there was a reverse trend in which WT mice accomplished superior results. No differences between individual genotypes were demonstrated in mice undergoing open field and EPM test after 30 days since the injury. At the same time, the results of rota-rod and MWM tests were poorer in CX3CR1^GFP/GFP^ mice compared to WT mice. The observation of the degree of neuronal loss with Fluoro-Jade B^+^ and NeuN^+^ staining demonstrated that CX3CR1^GFP/GFP^ homozygotes lost fewer neurons in the acute phase in comparison with WT mice. However, in the chronic phase, the trend was reversed. The analysis of cytokine level showed the reduced expression on days 7 and 15 after CCI for IL-1β, reduced IL-4 and IL-6 on day 15, and reduced iNOS on day 7. No changes were observed as regards the expression of transforming growth factor beta (TGF-β) on day 7 following the injury. The authors, just like Zanier et al., suggested that the inhibition of CX3CL1/CX3CR1 axis conduction may be a potential therapeutic target in the acute phase after an injury basing on the analysis of the studied parameters and the activity and proliferation of microglia, observing the CD11b^+^ cell count on days 15 and 30 after the injury and the kinetics of the expression of superficial markers for specific subtypes of activated microglia (YM1, CD206, CD68, and MARCO) within the studied tissues. Regarding the immune response occurring in case of ICH which is a common consequence of TBI, we need to mention the findings of Taylor et al. [183]. They used chimeric CX3CR1^GFP/GFP^ mice in which ICH was modeled via the injection of whole blood into the striatum. It was demonstrated that in this model, CX3CR1 deficiency did not affect monocyte recruitment, functional recovery, or immune response manifested as the changes of cytokine expression levels.

**Table 3:** Observational studies implicating the role of local and systemic immune response in the context of CX3CL1/CX3CR1 axis functioning in traumatic central nervous system injury

Reference	Model	Animal	Effect on monocyte recruitment	Post-injury regulation of expression								Clinical outcome
				IL-1β	IL-6	IL-4	IL-10	TNFα	iNOS	TGF-β	IGF-1	
Donnelly et al. [162]	SCI	CX3CR1^GFP/GFP^ mouse	↑	−	↓			−	↓			Improvement in BMS
Blomster et al. [164]	SCI	CX3CR1^GFP/GFP^ mouse	↑									Worsening in BMS
Blomster et al. [178]	OBX	CX3CR1^GFP/GFP^ mouse	↑	↑	↑			↑				
Morganti et al. [179]	TBI	CX3CR1^GFP/GFP^ mouse		↓	↓			↓	↓			Improvement in RAWM test
Zanier et al. [180]	TBI	CX3CR1^−/−^ mouse	↑/↓	−			↑	−	↑/↓		↑/↓	First improvement and then worsening in neuroscore test
Febinger et al. [182]	TBI	CX3CR1^GFP/GFP^ mouse	↑/↓	↓	↓	↓			↓	−		First improvement and then worsening in neuroscore test
												No differences in open field and EPM test
												Worsening in rota-rod and MWM test
Taylor et al. [183]	ICH	CX3CR1^GFP/GFP^ mouse	−		−			−				No differences in cylinder test, open field, and forced run test

*SCI* spinal cord injury, *OBX* olfactory bulbectomy, *TBI* traumatic brain injury, *ICH* intracranial hemorrhage, *BMS* Basso Mouse Scale, *RAWM* radial arm water maze, *EPM* elevated plus maze, *MWM* Morris water maze; ↑ increase in mentioned variable, ↓ decrease in mentioned variable, − without change in mentioned variable

## Potential Therapeutic Options

**Fig. 4:**
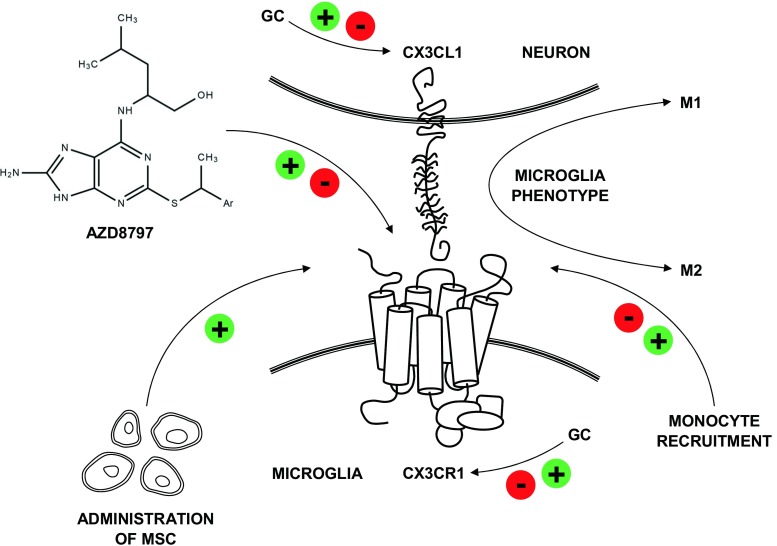
Overview and summary of potential CX3CL1/CX3CR1 axis-associated therapeutic options for management of traumatic brain and spinal cord injury. *GC* glucocorticoids, *MSC* mesenchymal stem cells; (+) refer to activation and (−) to inhibition of CX3CL1/CX3CR1 axis by acting on protein expression and CX3CL1/CX3CR1 interaction

The analysis of the literature shows that the possibility of linking CX3CL1 and CX3CR1 concentrations with such prognostic factors as BBB dysfunction, ICP, and GCS seems particularly promising in the clinical aspect [156, 159]. Regrettably, the scarcity of studies conducted so far make it impossible to determine the details of the functional role of CX3CL1 and CX3CR1 in the pathomechanism of TBI and SCI and unambiguous qualification of this signaling axis as neuroprotective or neurotoxic. According to the literature describing the mouse model of KO CX3CR1^GFP/GFP^ homozygotes, the inhibition of CX3CL1/CX3CR1 axis conduction contributes to an intensified recruitment of monocytes at the injury site in the majority of cases, which leads to varied effects as regards the expression of other cytokines (IL-1β, IL-6, TNFα) and iNOS. Therefore, scoring and testing bring heterogeneous results of the cognitive and motor neurological functions of the evaluated animals. The discrepancies in the interpretation of study results and a paucity of research on patients result from differences in the modeling of animal experiments and the difficulty obtaining uniform patient groups qualified for the study, because CNS injuries occur unexpectedly and are a highly heterogeneous group of injuries. The present authors believe that it is necessary to conduct multicenter research comprising larger patient groups and to analyze other parameters, such as concomitant inflammatory diseases or patients’ drug history, which may significantly contribute to overcoming the difficulty obtaining more reliable results. According to the current state of knowledge about CX3CL1 and CX3CR1 both as regards physiological and pathological CNS conditions, it is still fundamental and valid to determine the role of this signaling axis precisely in individual medical conditions and to search for a proper therapeutic approach. It implies a distinct necessity to seek new types of immunomodulatory therapies. It seems that inhibiting more than one chemokine signaling axis in TBI, SCI, and other neuropathologies might have a positive effect on the specificity of obtained results and provide researchers with new data concerning the role of chemokines in the CNS and cells of various immunophenotypes which infiltrate injured neural tissue. Seemingly, a factor which plays a particularly significant role may be the effect on the inflammatory environment developing at the time of injury via modulating the microglia cell phenotype in individual phases following the injury in order to direct the polarization of its activity toward the neuroprotective aspect [180, 182]. The use of a selective CX3CR1 antagonist (AZD8797) may be a promising treatment of TBI and SCI in this case [184, 185]. Moreover, if we bear in mind the fact that the migration of mesenchymal stem cells (MSCs) is also dependent on CX3CL1/CX3CR1 axis, then their potential administration, either intravenously or directly to the cerebral ventricles, with or without simultaneous inhibition of the CX3CL1/CX3CR1 axis in various periods since the injury, might contribute to the success of this type of therapy. However, additional research is necessary [186–191]. Glucocorticoid (GC) administration is another pharmacological modality in TBI and SCI, which has been very well-known and commonly used for many years. However, it is still disputable as regards the effectiveness and long-term consequences [192–195]. Regarding the influence of GC on CX3CL1/CX3CR1 axis functioning, it needs to be emphasized that GC administration may contribute to the reduction of mRNA expression both for CX3CL1 and also CX3CR1 [196]. It may be due to the effect of GC on IL-1β expression level or the activity of NF-κB transcription factor [196]. In the light of this analysis, it is difficult to state whether GC modulation of the CX3CL1/CX3CR1 axis is a neuroprotective or neurotoxic therapeutic option in case of CNS trauma. Regrettably, the effect of GC and other pharmacological agents on the CX3CL1/CX3CR1 axis in the course of TBI and SCI has not been widely discussed. A thorough analysis of the role of the CX3CL1/CX3CR1 axis, particularly in the context of TBI and SCI, and the possibility of its modulation with drugs and immunomodulatory treatment may bring tangible benefits associated with the understanding of this type of injury and the possibility of comprehensive influence exerted on the immune response in order to obtain superior clinical results (Fig. 4).

## Conclusions and Future Perspectives

Traumatic CNS injuries pose a significant problem and a challenge for the modern society due to their multidimensional character, necessary interdisciplinary approach, and the absence of efficient treatment options. More and more attention has been paid to phenomena occurring during the secondary injury, with a very important element being the local and systemic immune response associated with the migration of immune system cells and the secretion of cytokines and chemokines. The role of conduction related to the CX3CL1/CX3CR1 axis, which is analyzed in this paper, has been well-known in the context of physiological regulation between neurons and microglia, which confirms its position as one of the most important neurochemokines. However, its role has not been widely discussed as regards TBI and SCI. According to available professional literature, the expression of CX3CL1 and CX3CR1 undergoes dynamic regulation both in the course of TBI and SCI. The regulation occurs both at the transcriptional and post-translational levels which results from the changes in the levels of CX3CL1 and CX3CR1 proteins and dedicated mRNA transcripts. The general tendency as regards mRNA expression indicates that CX3CR1 undergoes regulation to a larger extent than CX3CL1 which seems to be more dependent on the regulation at the post-translational level. The post-translational expression of CX3CL1 may be conditioned by the following factors: BBB and BSCB dysfunction, proteolytic cleavage by ADAM10 and ADAM17 proteases, rapid binding to CX3CR1, and penetrating between three compartments including the extracellular space, CSF, and circulating blood [156, 197, 198]. According to the majority of studies, the significant increase in the expression of CX3CR1 and its mRNA after an injury may be justified by the increased monocyte infiltration and increased activity and migration of microglia (CX3CR1^+^) and also in the local changes of the immunophenotype of these cells [162, 164, 178, 199, 200]. Moreover, it needs to be mentioned that there are potential limitations of fluorescent mouse model which was most commonly used in studies. The limitations are associated with the insertion of GFP protein in the CX3CR1 promotor which leads to haploinsufficiency and the reduced superficial expression of CX3CR1 in heterozygotes which are commonly used as control groups [201]. A significant aspect which impedes the understanding whether the influence of the CX3CL1/CX3CR1 axis is the same as regards the injuries of the whole CNS is the fact that there are substantial morphological and associated neuroinflammatory response differences between the brain and the spinal cord [46, 202]. It seems that in this case, the most important factors are the differences in the quantity, distribution and phenotype of microglia cells, permeability of BBB and BSCB after injury, and the quantity of recruited immune cells [46, 202]. Until now, the analysis of CX3CL1/CX3CR1 axis properties in other neuropathologies has not brought an unambiguous answer concerning its positive or negative consequences. The positive effects may include CX3CR1 deficiency in the course of ischemic stroke [203–205]. However, in conditions like Parkinson’s disease or amyotrophic lateral sclerosis (ALS), CX3CL1 deficiency is associated with the worst outcome [206, 207]. The analysis of CX3CR1 deficiency models in Alzheimer’s disease still leaves some ambiguity [208–210]. The analysis of professional literature demonstrates that CX3CL1/CX3CR1 axis is an attractive potential therapeutic target due to its properties of an inflammatory response regulator, which may facilitate the introduction of immunomodulatory therapies that, depending on the needs, may enable selective intensification or inhibition of an inflammatory response in the course of various neuropathologies with the final aim of obtaining superior clinical results. Thus, after discussing the structure and function of CX3CL1/CX3CR1 axis, the physiological function within the CNS, and its role in the course of TBI and SCI, it may be stated that in spite of a variety of existing and available studies, it is still necessary to conduct further multicenter-detailed research to determine the accurate location of this signaling axis in the pathogenesis of secondary injury development and, consequently, to develop new possible therapeutic methods.
